# Combining Experience Sampling and Mobile Sensing for Digital Phenotyping With m-Path Sense: Performance Study

**DOI:** 10.2196/43296

**Published:** 2023-03-07

**Authors:** Koen Niemeijer, Merijn Mestdagh, Stijn Verdonck, Kristof Meers, Peter Kuppens

**Affiliations:** 1 Faculty of Psychology and Educational Sciences Katholieke Universiteit Leuven Leuven Belgium

**Keywords:** digital phenotyping, mobile health, mHealth, mobile sensing, passive sensing, ambulatory assessment, experience sampling, ecological momentary assessment, smartphones, mobile phone

## Abstract

**Background:**

The experience sampling methodology (ESM) has long been considered as the gold standard for gathering data in everyday life. In contrast, current smartphone technology enables us to acquire data that are much richer, more continuous, and unobtrusive than is possible via ESM. Although data obtained from smartphones, known as mobile sensing, can provide useful information, its stand-alone usefulness is limited when not combined with other sources of information such as data from ESM studies. Currently, there are few mobile apps available that allow researchers to combine the simultaneous collection of ESM and mobile sensing data. Furthermore, such apps focus mostly on passive data collection with only limited functionality for ESM data collection.

**Objective:**

In this paper, we presented and evaluated the performance of m-Path Sense, a novel, full-fledged, and secure ESM platform with background mobile sensing capabilities.

**Methods:**

To create an app with both ESM and mobile sensing capabilities, we combined m-Path, a versatile and user-friendly platform for ESM, with the Copenhagen Research Platform Mobile Sensing framework, a reactive cross-platform framework for digital phenotyping. We also developed an R package, named *mpathsenser*, which extracts raw data to an SQLite database and allows the user to link and inspect data from both sources. We conducted a 3-week pilot study in which we delivered ESM questionnaires while collecting mobile sensing data to evaluate the app’s sampling reliability and perceived user experience. As m-Path is already widely used, the ease of use of the ESM system was not investigated.

**Results:**

Data from m-Path Sense were submitted by 104 participants, totaling 69.51 GB (430.43 GB after decompression) or approximately 37.50 files or 31.10 MB per participant per day. After binning accelerometer and gyroscope data to 1 value per second using summary statistics, the entire SQLite database contained 84,299,462 observations and was 18.30 GB in size. The reliability of sampling frequency in the pilot study was satisfactory for most sensors, based on the absolute number of collected observations. However, the relative coverage rate—the ratio between the actual and expected number of measurements—was below its target value. This could mostly be ascribed to gaps in the data caused by the operating system pushing away apps running in the background, which is a well-known issue in mobile sensing. Finally, some participants reported mild battery drain, which was not considered problematic for the assessed participants’ perceived user experience.

**Conclusions:**

To better study behavior in everyday life, we developed m-Path Sense, a fusion of both m-Path for ESM and Copenhagen Research Platform Mobile Sensing. Although reliable passive data collection with mobile phones remains challenging, it is a promising approach toward digital phenotyping when combined with ESM.

## Introduction

### Background

One of the greatest challenges in social and behavioral sciences is to obtain reliable information about what people do, think, and feel during the course of their day-to-day lives. It is critical to learn more about this because it can aid in the prevention, diagnosis, and treatment of mental and behavioral issues. Until recently, the gold standard for collecting data in everyday life has been the experience sampling methodology (ESM)—also known as ecological momentary assessment (EMA)—in which ≥1 daily questionnaires are administered (nowadays via smartphones) to participants to report on their everyday behavior, thoughts, feelings, and context. However, the information that can be obtained through this method is limited owing to the nature of subjective self-report and participant burden. Nevertheless, recent advances in smartphones and other portable digital technologies allow us to collect much rich and more comprehensive information that goes well beyond what is available via ESM.

Prompting participants to complete multiple questionnaires per day, especially over long periods, can quickly become burdensome, resulting in deteriorating data quality or even participant dropout [1-3]. This problem may be partially mitigated in a research context because participants are generally motivated by monetary or other incentives, but it is more of a problem in clinical practice because the substantial participant burden makes it difficult to persuade individuals to use ESM on their own. Furthermore, although ESM has proven to be a substantial improvement over traditional questionnaires that only retrospectively inquire about past experiences (whereas ESM generally focuses on the present), the method’s reliance on self-report and inherent subjectivity can influence data quality through participants’ biases in terms of self-representation, introspective capabilities, and memory [1,3,4].

However, smartphones can not only administer questionnaires but can also collect all types of other information about the behavior, activity, and context of its user. These data are known as mobile or smartphone sensing data and include data about location, movement, activity, phone use, and app use, among others [5-7]. Mobile sensing data can contribute to research on what happens in people’s daily lives because it is able to track people’s behavior and environment unobtrusively, objectively, and without effort, thus revealing patterns that could not be discovered until now. Mobile sensing and other passive sensing methods have become increasingly important in the era of digital phenotyping [8], which is defined as “moment-by-moment quantification of the individual-level human phenotype in situ using data from personal digital devices” [9]. The use of digital phenotyping and mobile sensing has seen a huge increase, yet it has not been able to fulfill its promise to provide directly meaningful insight into people’s thoughts and feelings [10]. Given that ESM and mobile sensing each have advantages and disadvantages, a possible path forward is to complement ESM with mobile sensing to maximize the opportunities of both methods (and minimize their weaknesses).

To do this, a mobile app that is capable of collecting both ESM and mobile sensing data in the background must be used. Although there are some mobile apps that are already available (eg, AWARE [11]; mind Learn, Assess, Manage, and Prevent [12]; Beiwe [13]; Remote Assessment of Disease and Relapse–Base (*RADAR-Base*) [14]; Sensus [15]; and Effortless Assessment of Risk States [16]), most other apps are no longer maintained or poorly documented, posing a major barrier for researchers who want to incorporate mobile sensing into their research. In addition, most of the existing mobile sensing apps are focused on passive data collection and pay relatively little attention to complementing the obtained data with ESM, which could be a strong addition for examining many of the phenomena under study. For example, although location via GPS is able explain some of the variation in depressive symptoms, such symptoms have been linked more strongly to the emotions that people report to experience from moment to moment (using ESM) [17,18]. The limited predictive power of mobile sensing data is exacerbated in attempts to predict variables that change even more quickly such as momentary mood. In conclusion, although mobile sensing data can provide some information on participants’ everyday behavior, thoughts, feelings, and context, its stand-alone usefulness is limited when not combined with other sources of information such as ESM.

In particular, there are several ways in which ESM and sensing data can complement each other [19]. First, mobile sensing can add information to ESM measures such as biological, behavioral, or contextual variables of interest that cannot be reasonably measured by using only ESM. For instance, weather information based on participants’ current location can add to the understanding of stress [20] and well-being [21-23]. Second, mobile sensing could substitute or corroborate ESM measures by replacing items that can be measured directly with mobile sensing. For example, instead of asking participants where they are, the item could be replaced by mobile sensing that unobtrusively tracks their continuous location via GPS. Although this can also be measured with ESM, it is more subjective as opposed to real-world measurements from the phone itself, while also operating at a much higher sampling frequency. Third, it could improve the precision of ESM measures by allowing for event-contingent sampling or context-aware triggering [19]. An example of this is geofencing, a type of location service that can trigger a survey if the smartphone enters or exits a predetermined perimeter (eg, 50-m radius around their home). Finally, mobile sensing can also play an important role in ecological momentary interventions [24] and just-in-time adaptive interventions [25], where real-time data collection can trigger interventions or investigation of the participant’s condition. For example, evaluation of their position via GPS can monitor movements (eg, going out of the house) or certain high-risk locations (eg, liquor stores) and launch a prompt with specific questions or instructions.

### Objectives

To facilitate the complementary use of ESM and mobile sensing data for researchers, we created a new platform (by integrating 2 existing platforms) to enable the combined collection and application of ESM and sensing data. In this paper, we present a novel, full-fledged ESM platform with background mobile sensing. This new platform was evaluated based on 2 key criteria: the sampling reliability of mobile sensing and the perceived user experience of the technical implementation (battery drain, bugs, etc). First, we describe how this platform was created, its sensing capabilities, privacy and security considerations, general workflow for end users, and data processing and visualization for researchers. The second part of this paper contains the findings of a pilot study that used the new platform to evaluate the sampling frequency and user experience criteria.

## Methods

### Implementation

To develop an app with both ESM and mobile sensing capabilities, we used m-Path [26] as the starting point, as it is well established in both research and clinical settings. m-Path is a versatile and user-friendly platform for ESM and ecologic momentary interventions that has already proven itself as an asset to >15,000 users. Since 2019, >500,000 questionnaires have been completed on the platform. Some of its advantages include its ease of use through the user-friendly web-based dashboard, its wide array of question types, the ability to create applets, and the highly tailorable control flow within questionnaires in which one can even use piped text and real-time computations within and between interactions. As we wanted to incorporate mobile sensing into m-Path, we named the new app *m-Path Sense*. m-Path is aimed at both clinical practitioners and researchers, whereas m-Path Sense is only primarily directed at researchers who want to conduct ESM studies using mobile sensing.

To enhance m-Path with mobile sensing functionality, we added the Copenhagen Research Platform Mobile Sensing (CAMS) framework, a reactive cross-platform framework for digital phenotyping [27]. CAMS is specifically designed to be integrated into other apps, acting as a loosely coupled component of the overall app. As both m-Path and CAMS are programmed in Flutter (Google LLC) [28]—a cross-platform programming framework that compiles to both Android and iPhone Operating System (iOS) apps—there is a unique opportunity to combine the 2 frameworks. Therefore, m-Path Sense has been made available in the Apple App Store [29] and the Google Play Store [30].

The integration of m-Path and CAMS was accomplished by adding the various CAMS components to m-Path as a plug-in and using them as needed. A first trigger point for CAMS is when the app is launched and when a beep (ie, a notification for a questionnaire) is pressed if the app was previously closed inadvertently. The CAMS pipeline is then activated as a result of this interaction. CAMS’s underlying code (written in Dart) creates a unifying interface for both Android and iOS, but this interface is then converted to a platform-specific code (ie, Swift, Java, or Kotlin).

Another crucial step in the integration of m-Path and CAMS was the configuration and validation of the various sampling schemes and study protocols. A sampling schema in CAMS defines a specific configuration of a sensor, such as the frequency at which the measure should collect data. The study protocol specifies which sensors should be used and how frequently they should be activated. Some sensors provide a stream of data; thus, they only need to be triggered once (for example, if it is triggered by an event), whereas others provide data only once; therefore, the period over which this should occur must be specified. Although CAMS provides some standard values, testing different values is a time-consuming yet crucial process because it can have a considerable impact on both the quality and quantity of data and on the participants’ smartphones. Moreover, it also provided an initial idea of how well CAMS qualitatively performed under different sampling conditions.

**Figure 1:**
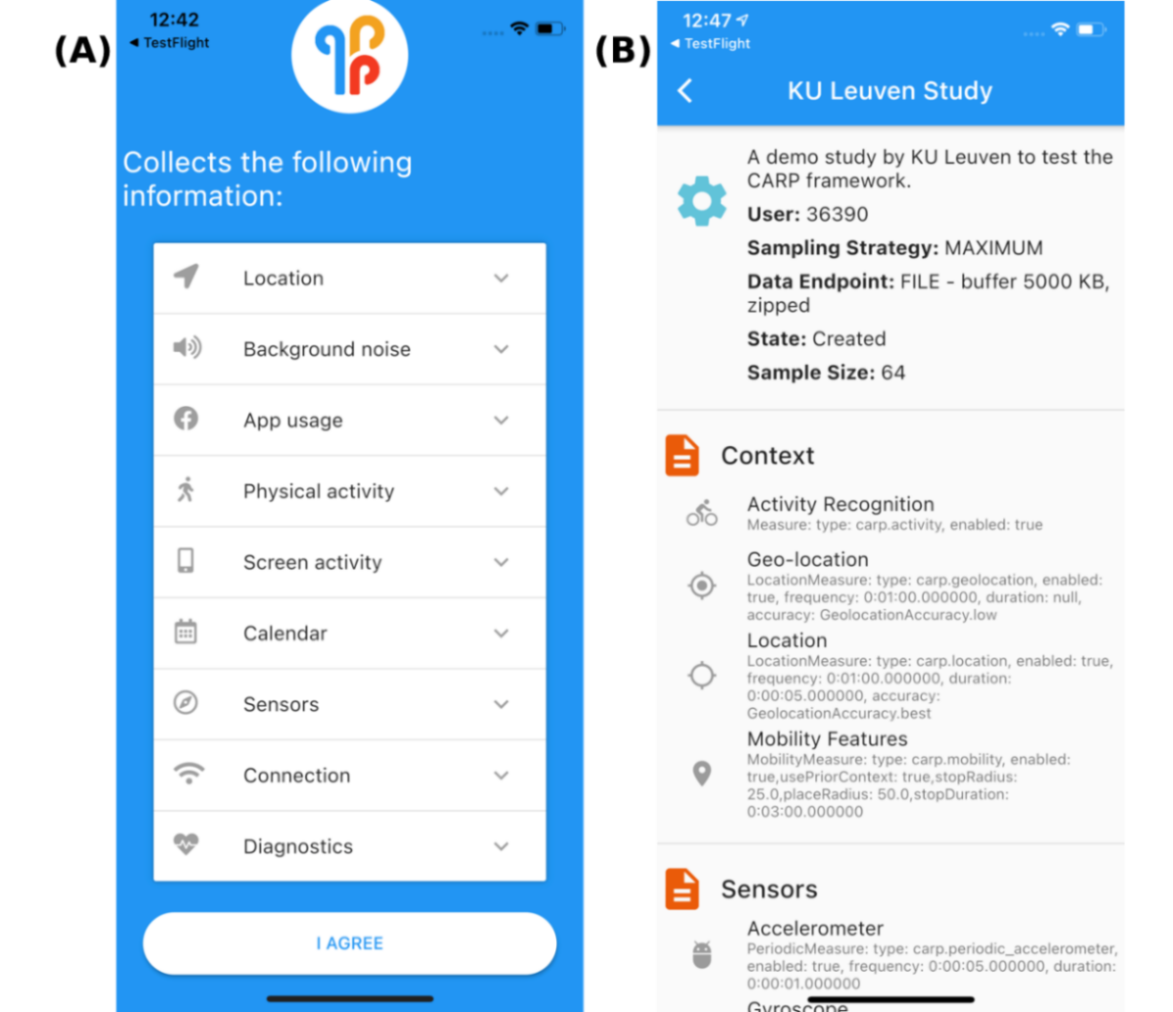
The newly developed app m-Path Sense was created by merging m-Path and Copenhagen Research Platform (CARP) Mobile Sensing. (A) The m-Path Sense introduction screen, which informs participants about what is being collected while also prompting the necessary permissions. (B) A detailed overview screen (adopted from CARP Mobile Sensing [27]) that shows what types of data are being collected (and how frequently). KU: Katholieke Universiteit.

The final step in the integration was to deal with the permissions (eg, location access) needed for CAMS (and parts of m-Path) to work properly. When participants first launch m-Path Sense, they must grant all permissions via a special screen that informs them about this requirement (Figure 1). These permissions are displayed individually to the participants. Currently, it is necessary to grant all permissions for the app to function properly, but we intend to customize this process based on which sensors are included in a particular study in a future version.

### Mobile Sensing Functionality

m-Path Sense is capable of collecting a wide array of mobile sensing data, depending on whether this type of data is available on the device’s operating system (OS; ie, Android or iOS). Table 1 lists all the available sensors responsible for capturing their corresponding data. It should be noted that the functionality for collecting call and text logs is not listed in Table 1 but is supported (for Android only). However, as collecting these types of data is against Google’s policy, they are not included in the Google Play Store version.

**Table 1:** Overview of available sensors in m-Path Sense and their corresponding default sampling frequency.

Sensor	Description	Android	iOS^a^	Default sampling frequency	Remarks
Accelerometer	Acceleration force along the x, y, and z axes (including gravity), measured in m/s^2^	Yes	Yes	Multiple times per second	Depends on the operating system but generally varies from 5-50 times per second
Activity recognition	Discrete activity label from Google’s or Apple’s activity recognition algorithm	Yes	Yes	When activity changes	N/A^b^
Air quality	Live air quality index from the nearest station	Yes	Yes	Every hour	N/A
Ambient noise	Volume (in decibels) of ambient noise; no audio is saved	Yes	No	Every 5 minutes	Code for iOS is present but not yet operational
App use	List of used app names and duration of use since the last measurement	Yes	No	Every 30 minutes	API^c^ is not available on iOS
Apps	List of installed apps on the participant’s device	Yes	No	Every day	API is not available on iOS
Battery	Current battery level	Yes	Yes	When battery level changes	On iOS only; triggers when charging state changes
Bluetooth	Scan of Bluetooth device in the vicinity	Yes	Yes	Every 5 minutes	N/A
Calendar	Information about the individual’s calendar appointments (eg, location and time)	Yes	Yes	Every day	N/A
Connectivity	Current connectivity status (ie, mobile data or Wi-Fi)	Yes	Yes	When connectivity changes	N/A
Device	General device information (model, manufacturer, etc)	Yes	Yes	When app restarts	N/A
Gyroscope	Rate of rotation along the x, y, and z axes, measured in radians per second	Yes	Yes	Multiple times per second	Depends on the operating system but generally varies from 5-50 times per second
Light	Light intensity, measured in lux	Yes	No	Every minute	API is not available on iOS
Location	Fused location estimate (GPS coordinates) stored using Curve25519 public key encryption	Yes	Yes	Every minute or when location changes	N/A
Memory	Free physical and virtual memory of the device	Yes	No	Every minute	API is not available on iOS
Mobility	Calculated mobility features, such as entropy, location variance, and the number of meaningful places	Yes	Yes	On the basis of researcher-specified parameters	When user changes their position by a certain distance
Pedometer	Number of steps according to the phone’s built-in pedometer	Yes	Yes	When step count changes	N/A
Screen	Time stamp of screen on, off, and unlock events	Yes	No	When screen changes	API is not available on iOS
Weather	Live weather information (humidity, precipitation, etc) from the nearest weather station	Yes	Yes	Every hour	N/A
Wi-Fi	SSID^d^ and BSSID^e^ of connected Wi-Fi network (if any)	Yes	Yes	Every 10 seconds	N/A

^a^iOS: iPhone Operating System.

^b^N/A: not applicable.

^c^API: application programming interface.

^d^SSID: service set identifier.

^e^BSSID: basic service set identifier.

### Security and Privacy

First, m-Path Sense is a completely separate app from m-Path to prevent users from inadvertently using m-Path Sense (both ESM and mobile sensing) instead of m-Path (ESM only) without being informed. On registration, they must also provide a code from a practitioner who has indicated in m-Path’s web-based dashboard that they allow mobile sensing functionality to use m-Path Sense. After providing informed consent and the sensing has started, participants can always stop sensing by closing or removing the app. There is purposefully no pause button, as participants may accidentally press it and consequently stop sensing inadvertently. At the same time, sensing data can only be gathered as long as the app is running; therefore, participants can always choose to terminate it or even remove the app entirely if they feel that their privacy is at risk. As long as the sensing component is active, a permanent notification (Android) or a blue dot (iOS) is displayed to remind participants that they are being tracked. By going to the menu and pressing the *mobile sensing* tab, participants can also see their personal participant ID, specific sensors that are being run, and amount of data that has been collected.

Given the highly sensitive nature of collected data, data security and privacy are of utmost importance. These elements were maximally considered in the app development process and handling of data. All data collected with the m-Path Sense app (both ESM and sensor data) are initially stored locally in a protected folder on the participant’s smartphone, which can only be accessed through the app. This folder cannot be accessed by other apps. Furthermore, we have implemented a privacy scheme [27] that can render certain extrasensitive data unreadable by using a 1-way cryptographic hash or encrypting it with an asymmetric Curve25519 public key [31]. Managing the storage of the private key is the responsibility of the researcher, such that the m-Path Sense team will never have access to the encrypted data in transit.

For the transfer of data from smartphone to server, asymmetric HTTP Secure encryption is in place. The server used in this study was owned by the university, but we are currently migrating to pCloud [32], a secure Europe-based cloud storage service [33,34]. Researchers then grant the app access to their own pCloud folder, and the app can directly upload data to their folder via HTTP Secure, from which the encrypted data can be downloaded. To enhance data security and to prevent data leakage, highly secured application-layer encryption is applied at all times. Specifically, all answers given to questionnaires are stored on the phone using Advanced Encryption Standard (AES) 256-bit encryption with Public-Key Cryptography Standard (PKCS) 7 padding. The collected mobile sensing data are written to a JSON file until this file has reached a size of 5 MB and subsequently zipped to reduce its size to approximately 1 MB. Both questionnaire and sensor data are transferred to secure servers only if the participant has access to a Wi-Fi network. In the rare event when participants do not have access to a Wi-Fi network for longer than 24 hours, data are uploaded via their mobile data connection or they can always upload the data manually at any other time. The data stored in this local folder are automatically deleted once it is sent to the server. Moreover, all local data are deleted once the app is removed from the phone.

### Processing and Visualization

In the current implementation, the data from the participants’ smartphones are stored as JSON files of up to 5 MB. On completion of each beep, all data (if connected to Wi-Fi) are sent to a secure server. This means that (1) data arrive in batches (and not in real time) and (2) the data are not immediately structured and integrated with all other data. In other words, the data will first have to be extracted from JSON files (where sensor data are also not written in sequence) and then imported into a central database.

To aid in the process of data processing, we have developed and made available an R package on the comprehensive R archive network (CRAN), named *mpathsenser* [35]. The central function in this package is called *import*, which reads the JSON data, extracts it, and writes it to an SQLite database. Although other database systems are more powerful and possibly fast, we opted for SQLite because it is widely available, can be easily shared offline (because it is only a single file), and can be automatically installed in R with the *RSQLite* package [36].

One of the specific challenges in analyzing mobile sensing data alongside ESM data is that the 2 must be aligned, despite being collected at very different timescales and frequencies [37]. The function *link* in the *mpathsenser* package does exactly this. It allows the user to link 2 tables together within a certain time window, for example, 30 minutes before or after each beep. Another issue that the R package assists in overcoming is the fact that mobile sensing data are often large in size, making efficient analysis difficult. Thus, many functions in the R package are written in such a way that they are executed in the SQLite database (and the computations in SQL), and only the result of the computation is returned to R.

**Figure 2:**
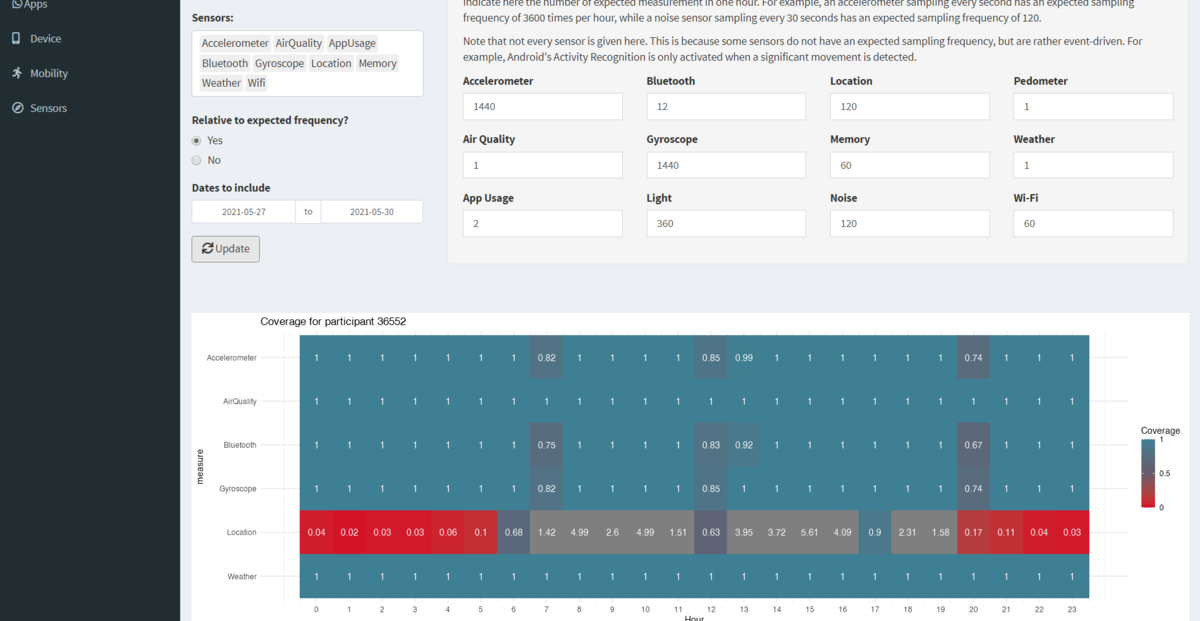
A screenshot of the R Shiny dashboard for keeping track of the data. In this coverage chart, the numbers indicate the ratio of the actual number of measurements to the expected number of measurements.

Furthermore, a dashboard built in R using Shiny [38] has been made available, allowing the researcher to import new data with a few clicks and visualize it in various ways to assess data quality. For example, a coverage chart (Figure 2) can be generated for a user that displays the measurement frequency per sensor, which can be used to inspect the eventual data collection frequency on particular smartphones, check whether the app has stopped working at some point, or identify any underperforming sensors.

### Workflow

After installing m-Path Sense, the workflow is as follows. When participants start m-Path Sense for the first time, they are prompted with a screen that informs them about every type of data that is being collected (Figure 1A). If they want to continue, they can press “I agree” and are subsequently prompted with a series of in-app permission modals where they have to grant access for m-Path Sense to be able to collect sensor data. After that, they have to agree with m-Path’s terms and conditions to continue and enter the researcher’s ID code (which is usually provided during the briefing session) to identify the person who has access to the collected data. This code also helps to prevent unintentional sign-ups by people who download the app while not participating in any study. The app will start data collection only after these steps are completed. After the study has started, participants can always view which data are being collected (Figure 1B) and withdraw from a study at any time by removing the app.

### Pilot Study

To evaluate the app, we conducted a 3-week pilot study in which we administered ESM questionnaires while mobile sensing data were being collected in the background. In addition to answering substantive questions about the value of digital phenotyping for psychological variables, this pilot study served to evaluate the app based on several criteria. First, we aimed to evaluate the reliability of the sampling and its related ability to stay alive on the participant’s phone as this is a crucial challenge with mobile sensing apps. A well-known problem for mobile sensing apps is that OSs have become increasingly strict in pushing apps further to the background and eventually stopping them; thus, a crucial challenge for such apps is to enable sufficient data coverage [39-41]. Second, we wanted to evaluate the perceived general user experience of the app, that is, whether participants had any trouble with battery drain, app crashes, or other nontechnical aspects. The ease of use of the ESM system was not evaluated, as m-Path has already been widely used, evaluated, and tailored [26].

Between June 2021 and December 2021, an initial pool of 462 participants was recruited through various Facebook groups and an experiment recruitment system associated with Katholieke Universiteit Leuven. On the basis of a prescreening process, we excluded participants who were (1) not native Dutch speakers, (2) aged <18 years, or (3) having a phone that was very old (older than Android 7.0 or iOS 13.0) to meet the technical requirements of the app. Participants were then selected based on their availability in the study period and their level of neuroticism (according to the relevant Big Five Inventory-2 [42,43] items regarding neuroticism) to achieve variability in emotional functioning. After applying these inclusion and exclusion criteria, 22.5% (104/462) of the participants were retained (men: 12/104, 11.5%; women: 91/104, 87.5%; other: 1/104, 0.9%; mean age 20.70, SD 4.08; range 18-52 years). An overview of the specific sampling schema used in this pilot study is presented in Table 2.

**Table 2:** Sensors and their sampling frequencies used in the pilot study.

Sensor name	Sampling frequency	Android	iOS^a^
Accelerometer	Every 5 seconds; 1 second of measurements	Yes	Yes
Activity	Event based	Yes	Yes
Air quality	Every 1 hour	Yes	Yes
App use	Every 30 minutes	Yes	No
Battery	Event based	Yes	Yes
Bluetooth	Every 5 minutes	Yes	Yes
Calendar	Every day	Yes	Yes
Connectivity	Event based	Yes	Yes
Device	Every day	Yes	Yes
Gyroscope	Every 5 seconds; 1 second of measurements	Yes	Yes
Installed apps	Once per day	Yes	No
Light	Every 1 minute; 10 seconds of measurements	Yes	Yes
Location	Every 1 minute; also event based	Yes	Yes
Memory	Every 1 minute	Yes	No
Mobility	Event based	Yes	Yes
Noise	Every 1 minute	Yes	No
Pedometer	Event based	Yes	Yes
Screen	Event based	Yes	No
Weather	Every 1 hour	Yes	Yes
Wi-Fi	Every 10 minutes	Yes	Yes

^a^iOS: iPhone Operating System.

All volunteers provided their informed consent after receiving information regarding the study protocol, remuneration, and obligations and advantages of participation. Participants were advised that their participation was entirely voluntary and that they could exit the study at any time. To preserve the privacy and confidentiality of participants, the data used in this study were pseudonymized. Personal information is kept separate from study data and will not be shared with third parties. Furthermore, as mentioned in the *Security and Privacy* section, several procedures were implemented to assure data security. Participants were reimbursed for their time through university course credits or monetary compensation of up to €70 (US $79). The remuneration was dependent on the rate of compliance with the questionnaire responses. Participants received full remuneration of €70 (US $79) or 10 credits if they met a compliance rate of at least 75% of the responses on the ESM questionnaires. Each 10% reduction in compliance resulted in a reduction of €10 (US $11.29) or 1 credit of compensation.

### Ethics Approval

This study was approved by Katholieke Universiteit Leuven Social and Societal Ethics Committee (G-2020-2200-R3[AMD]) and was conducted in compliance with human subject research ethical principles.

## Results

### Overview

On the basis of the results of the pilot study, this section describes the processing of the data output (ie, the raw data); the data quality in terms of sampling reliability; and finally, the participants’ perceived user experience regarding the functionality of the app but not its design. By doing this, we evaluated 2 key criteria, namely, whether the sampling frequency was satisfactory and sufficiently reliable and the perceived user experience. Regarding platform differences, the pilot study sample included 50% (52/104) iOS devices and 50% (52/104) Android devices. The most common brands of phone were Apple (52/104, 50%), Samsung (28/104, 26.9%) and OnePlus (10/104, 9.6%). Other brands included Nokia (2/104, 1.9%), Motorola (1/104, 0.9%), OPPO (1/104, 0.9%), Realme (1/104, 0.9%), and Xiaomi (1/104, 0.9%).

### Data Output

A first step toward evaluating the data from m-Path Sense is to examine the raw data output in the form of either JSON or ZIP files. It should be noted that the ZIP files contain JSON files also, but that JSON files may appear in the data output when they were not properly closed because, for example, the app was killed. To avoid JSON syntax errors, when the app is restarted, it will begin writing to a new JSON file rather than continuing to write to the previous one. When this occurs, the old JSON file usually has incorrect file endings, indicating that it is not in the valid JSON format. One of the functions of the R package, *mpathsenser*, is to automatically fix this. After unzipping the data, 5.55% (4654/83,875) of the files had to be repaired, including some (approximately 50/4654, 1.01%) that were partially corrupted and had to be repaired manually by simply deleting the corrupted parts in the file. It is unknown what causes file corruption, but it is most likely a problem with the participants’ phones (because most corrupted files that could not be fixed automatically belonged to a single user, 16/20, 80%) or a bug in Flutter’s internal file writing software.

In total, there were 5.51% (4622/83,875) JSON files and 94.49% (79,253/83,875) ZIP files across 104 participants, measuring 69.51 GB in size or 37.50 files and 31.10 MB per day per participant. Interestingly, iOS devices provided many more JSON files (140.12 MB) and ZIP files (921.42 MB) per person than Android (60.13 MB and 212.23 MB, respectively). This is also reflected in the time it took to fill an entire file (maximum of 5 MB); iOS devices needed a median time of 8.83 minutes before starting a new file, whereas Android devices took 1.80 hours before a new file was needed. The primary reason for this discrepancy is that iPhones produce far more accelerometer and gyroscope measurements than Android phones. In conclusion, even if a participant does not upload their data to the server for an entire day, only approximately 59.49 MB is stored on iPhones and 17.33 MB is stored on Android phones.

After the data are sent to the server, they are removed from the phone so that the file size is no longer an issue for users. However, for researchers, the file size of the (unpacked) data is still important owing to hardware constraints. The size of these data deviates from the previously stated figures because these were mainly ZIP files with highly compressed packed data. Extracting the ZIP files to JSON and then converting them into another format (for example, in an SQLite database) has consequences for the corresponding size. The total size after extracting the ZIP files (which only leaves JSON files) was 430.43 GB. Differences existed between Android and iOS devices. Android users, for example, had an average of 389 JSON files with an average size of 4.71 MB, whereas iOS users had an average of 1207 JSON files with an average size of 4.91 MB.

As the unpacked data were relatively large (430.43 GB) and parsing each file to read it in an SQLite database would further increase the size, importing all data to an SQLite database would be a time-consuming and computationally expensive task. However, the accelerometer and gyroscope sensors produced many observations per second, accounting for approximately 90% of the data (in terms of size). Consequently, reducing the data from these sensors while retaining relevant information would be conducive to performing analyses more efficiently. Using multiple values per second, we calculated the Manhattan distance (L1-norm); Euclidean distance (L2-norm); and average of each x, y, and z dimension per second, because for most purposes, 1 accelerometer or gyroscope value per second suffices. We used an incremental approach, by importing accelerometer and gyroscope data in chunks of about 60 GB and then shrinking the size by calculating these summary metrics. The total SQLite database size—including relevant indexes—after importing all data was 18.30 GB. Table 3 shows the number of observations.

**Table 3:** The number of observations per sensor across 104 participants over 21 days of sampling. It should be noted that these are the number of observations for the accelerometer and gyroscope after binning these values to once per second, greatly reducing the size of the data (N=84,299,462).

Sensors	Observations, n (%)
Accelerometer	29,464,849 (34.95)
Activity	6,443,664 (7.64)
App use	393,440 (0.47)
Battery	1,889,310 (2.24)
Bluetooth	1,989,703 (2.36)
Calendar	15,770 (0.02)
Connectivity	58,716 (0.07)
Device	4652 (0.01)
Gyroscope	26,896,404 (31.91)
Installed apps	113,256 (0.13)
Light	757,034 (0.90)
Location	3,675,559 (4.36)
Memory	743,018 (0.88)
Noise	382,771 (0.45)
Pedometer	3,397,663 (4.03)
Screen	397,726 (0.47)
Weather	23,793 (0.03)
Wi-Fi	7,649,719 (9.07)

### Sampling Reliability

**Figure 3:**
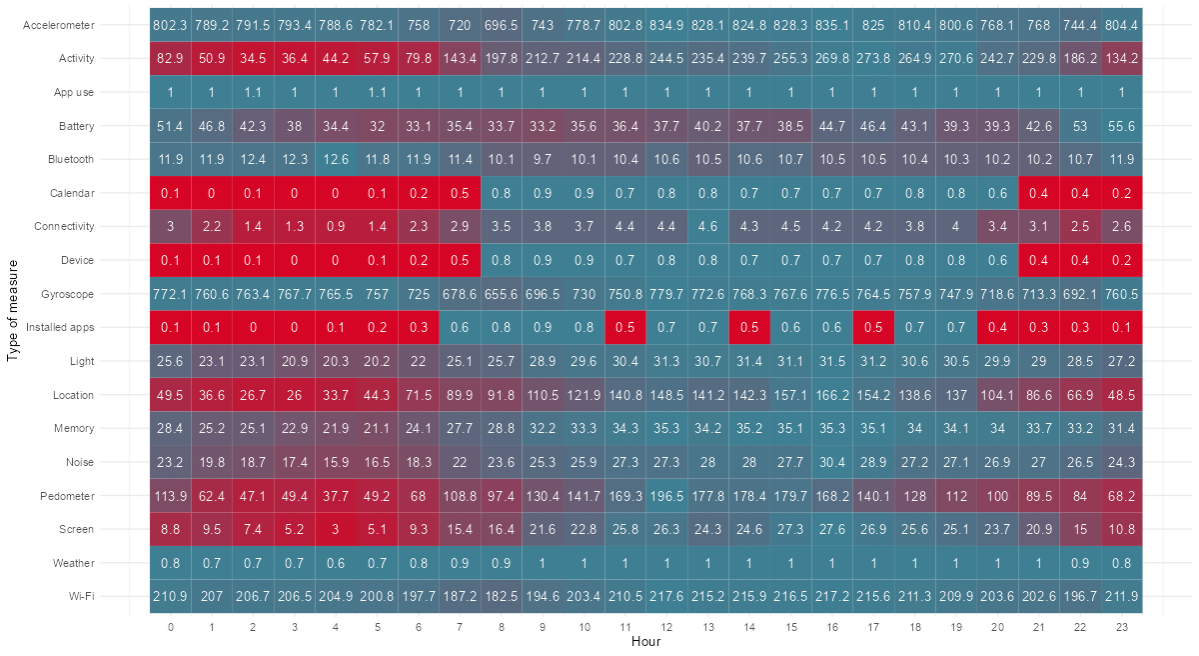
The average absolute number of measurements per hour across all participants, colored by frequency per sensor. The color range per row varies because the sensors measure at different frequencies. The scale’s lower bound is always 0 and completely red. The maximum observed sampling frequency for that sensor determines the upper bound. For example, weather has a value of 1 and is thus completely blue. However, in the case of location, it is only at 166.50 that it is completely blue, with approximately 1 measurement every 22 seconds. It is also worth noting that the accelerometer and gyroscope measurements were binned to an average value per second.

The first objective of the pilot study was to assess the reliability of m-Path Sense in terms of the sampling frequency, that is, whether the number of data points was satisfactory. Figure 3 depicts the average number of observations per hour for all participants. As separate sensors have different target sampling frequencies (and thus, different scales in the figure), the color range of each row is determined from the sensor’s sampling frequencies, ranging from 0 (red) to the maximum observed sampling frequency for that sensor (blue). In general, we found the number of collected observations for most sensors to be quite satisfactory in terms of providing sufficient data for most types of analyses and typical research questions. For example, the accelerometer sensor provides approximately 780 samples per hour (after binning), which is slightly more than once every 5 seconds. Another example is the location sensor that provides a location update 97.20 times per hour on average (once every 37.04 seconds), with far more updates during the day (once every 31.65 seconds) than at night (once every 1.74 minutes), instead of its targeted sampling frequency of once per minute. In general, sampling appears to be decreasing at night, possibly owing to the OS pushing the app to the background.

**Figure 4:**
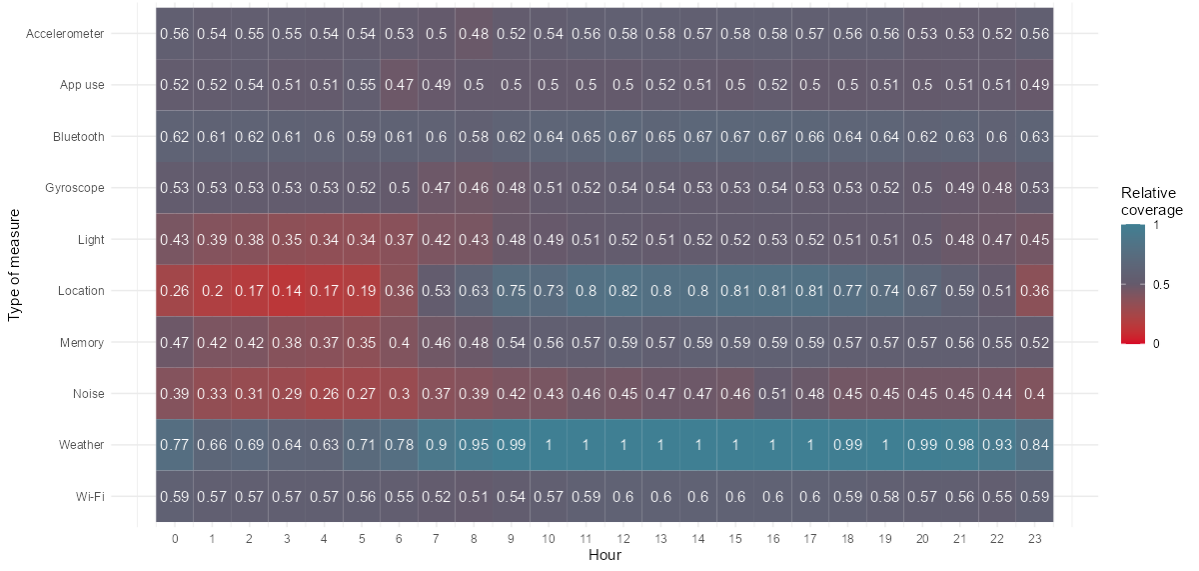
The relative number of measurements per hour averaged across all participants, where a value of 1 indicates that the actual number of measurements was exactly equal to the expected number of measurements and a value of 0.50 indicates that only half of the expected number of measurements were captured.

Although results when looking at the absolute number of measurements per hour appear to be good, the coverage rate—the ratio between the actual number of measurements and the expected number of measurements—may be a better method for assessing sampling reliability. Figure 4 depicts the pilot study’s relative coverage rate. Multimedia Appendix 1 provides an overview of the expected number of measurements, that is, the sampling schema. Figure 4 shows that the relative coverage is well below 1, frequently hovering around 0.50. This means that only half of the measurements were collected in comparison with what the app was designed to do. For example, if the location of the participants was supposed to be collected once every minute, it was only collected every 2 minutes. For most sensors, the targeted sampling frequency was quite high; therefore, even collecting half of the intended data may be sufficient for a given study. However, for a general-purpose mobile sensing app, this effect is generally undesirable.

**Figure 5:**
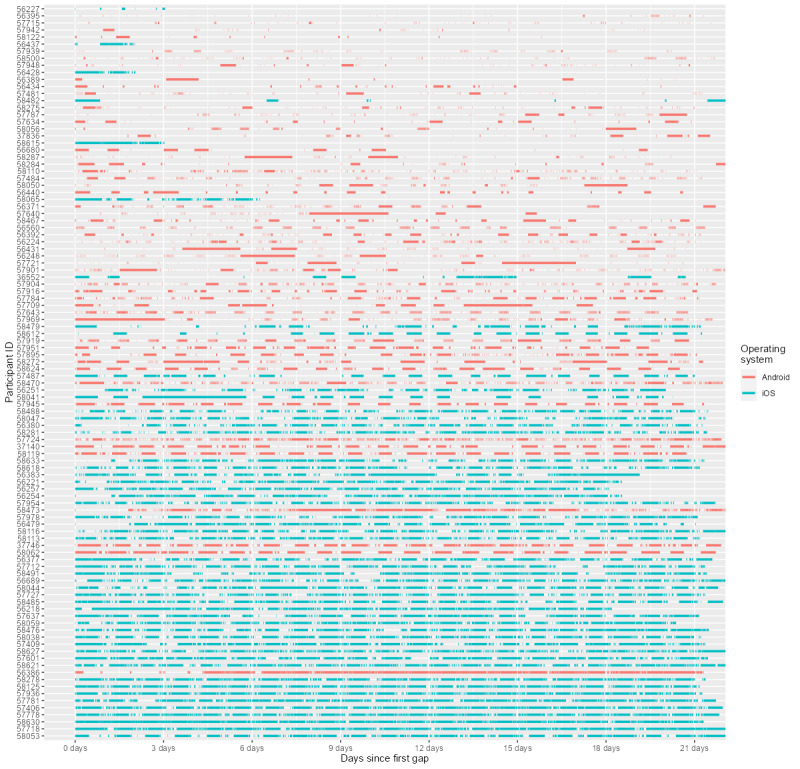
Gaps in the data over time per participant. Each colored bar represents a gap, as measured by the absence of accelerometer measurements for 5 minutes. iOS: iPhone Operating System.

The low relative coverage rate raises the question of why few observations were collected. A related issue is that the data contains a large number of gaps (Figure 5). In this case, a gap is defined as a period of at least 5 minutes during which no measurements were recorded by any sensor. Counterintuitively, the colored bars in Figure 5 depict these gaps over time for each participant. During the 21-day pilot study, Android users had a median of 157 gaps lasting 7.55 minutes in their data, whereas iOS users had a median of 165 gaps lasting 47.36 minutes. Thus, although Android and iOS users had roughly the same number of gaps in their data, gaps occurring on iOS devices were much long, resulting in great loss of data. Naturally, the more gaps there are in the data, the fewer observations can be collected: consider a total data loss of 4.19 days for Android users and 1.95 weeks for iOS devices, both of which would be undesirable. Fortunately, nightly data could account for a large portion of this data loss. After removing gaps between 12 PM and 6 AM, the total data loss per participant over the 21-day study period was 23.93 hours for Android devices and 5.02 days for iOS devices.

Furthermore, we also assessed whether there were differences between successive OS updates. To this end, we ran a 2-way ANOVA (*α*=.05) with the total gap time per participant as the dependent variable and whether it was a *new* or *old* version of this OS as independent variables. The classification was required because there may be only a few participants for some OS versions, resulting in severely unbalanced groups. For example, there were only 9.6% (10/104) participants who used an OS older than Android 10. iOS 15 (22/104, 21.2%) and Android 11 (30/104, 28.8%) were considered as *new* OS versions, whereas iOS 14 (22/104, 21.1%; there were no older versions) and Android 10 or lower (23/104, 22.1%) were *old* OS versions. Our ANOVA results suggested that devices running Android experienced significantly fewer gaps (mean 5.59, SD 4.01 days) than devices running iOS (mean 1.81 weeks, SD 4.97 days; *F*_1,102_=52.10; *P*<.001; η^2^ [partial]=0.34, 95% CI 0.22-1). Specifically, the results show that there was a difference in the total gap time between Android and iOS. However, there were no differences between *old* and *new* versions within either Android (old: mean 4.50, SD 3.87 days; new: mean 6.43, SD 3.98 days) and iOS (old: mean 1.68 weeks, SD 5.81 days; new: mean 1.99 weeks, SD 3.32 days; *F*_1,102_=3.69; *P*=.06; η^2^ [partial]=0.03, 95% CI 0-1). Therefore, although there were differences between Android and iOS in terms of the overall number of gaps, the specific OS versions did not show statistically significant differences within each OS.

One of the most likely causes of these data gaps is the OS itself, specifically how it attempts to save energy and resources when the device is not in use (eg, Android’s doze mode). There are some guidelines [44] that should help prevent the app from being gradually pushed into the background (and eventually killed), even though both Android and Apple have become increasingly strict in recent years on apps that consume battery in the background. A solution that is currently being implemented to mitigate this issue is to send a signal to the server every 5 minutes to show that it is still alive. When the server does not receive this signal, it can send a notification to the app, which the user must then click on to resume sampling.

In addition to the app simply being pushed to the background and eventually killed, we investigated several other possible causes for the gaps in the pilot study data. Differences between smartphone brands were one of the first explanations we considered. For example, it is possible that a particular brand may be underperforming, thus lowering the overall coverage rate. Multimedia Appendix 2 shows the average coverage rate per day for each brand. There are some differences between brands, but they are minor. A second hypothesis proposed that the low average sampling frequency was caused by a small number of participants who provided very little data. The average relative coverage per participant is presented in Multimedia Appendix 2. This demonstrates that, although there is some variation in the average relative coverage of participants for most sensors, there are no obvious outliers or skewed distributions. Finally, we hypothesized that the large gaps in iOS data were caused by a previously identified problem in the study, namely, iOS abruptly deleting some files owing to a backup issue. If the m-Path Sense folder became very large, they were *backed up* by iOS, which inexplicably deletes all files. After a few days, we fixed this bug by explicitly stating that these data should not be backed up and that it will be sent to the server more frequently. However, as this backup solution only applied to log-in data, the sensing data could still be compromised. A method to evaluate the impact of this problem would be to identify a relationship between the proportion of Wi-Fi and mobile data and the total number of gaps. If the data were sent every 5 minutes_ _when connected to Wi-Fi, the possibility of a large amount of data being lost is low. Thus, participants who use Wi-Fi more frequently will most likely have few gaps, and a negative association between total Wi-Fi time and gaps stands to reason. Although there was a negative relationship between the 2 aspects, it was neither very strong (*r*=−0.16) nor statistically significant (*P*=.10) when considering the OS version.

### User Experience

We assessed the participants’ perceived user experience with m-Path Sense using feedback from the debriefing session and an ESM item assessing app issues (“Has your smartphone and/or app worked normally since the last beep?”). The figures in parentheses represent the number of participants who mentioned the issue at least once; however, this was not a structured interview because we wanted to let the participants decide what they thought was most important to address. They were asked, among other things, whether they had any problems with the app, what parts bothered them, and what could be improved.

First, participants noticed minor battery drain (as also reported by battery tests [27]), but this was not perceived as bothersome (27/104, 25.9%). This is also reflected in the fact that only 1.34% (133/9924) of all beeps received on Android and 15.00% (1365/9096) of all beeps on iOS devices reported that participants noticed the battery running down faster than usual at that time. However, when asked whether this was a burden during the debriefing session, most participants (27/104, 25.9%) reported that it was manageable because they carried their charger with them.

Second, some participants (18/104, 17.3%) reported that the app sometimes crashed or froze on start-up; however, this was only reported in 2.23% (221/9924) and 0.45% (41/9096) of ESM beeps for Android and iOS users, respectively. Finally, participants who used an iOS device (12/104, 11.5%) mentioned a problem at the beginning of the study (for iOS only), where some of m-Path Sense’s files were deleted by the OS, causing the app to stop working. Participants who experienced this issue were asked to reinstall the app, and the problem was permanently resolved through an update after a few days.

## Discussion

### Principal Findings

In this study, we attempted to combine the collection of ESM and mobile sensing data in 1 app, m-Path Sense, and an associated R package and Shiny app, so that these data can be easily brought together and can interact with each other in the future. The platforms combined for this purpose are m-Path [26] and CAMS [27]. During integration, there was a strong emphasis on security and privacy, such as the decision to create a separate version for m-Path Sense (rather than including this as an option in m-Path itself) and encrypting and hashing different types of data.

A pilot study with 104 participants was conducted to assess the evaluation criteria (sufficient reliability and not very invasive for the user). Although the total amount of data collected was adequate for most studies, it was less than the intended sampling frequency, likely caused by the OS’s attempts to save energy and resources when the device is not in use (eg, Android’s doze mode). This issue is not uncommon in the mobile sensing literature. For example, a study [40] discovered that when collecting geolocation data using a smartphone, 12% of all gaps lasted longer than 60 minutes, even though measuring every 30 minutes was planned. In another study [39], 17.2% of participants had only 2 location measurements per day, whereas 24 were planned, and 17.2% had no measurements at all, with significant differences between Android (better) and iOS. As these gaps may have a direct impact on sampling reliability, it is critical to assess their effect on studies’ findings.

As the field of digital phenotyping and mobile sensing research expands and evolves, we can expect increased scrutiny and device OS constraints imposed by corporations such as Apple and Google. Further limitations may include strict guidelines for data collection, such as limitations on the types of sensors that can be accessed and prioritizing transparency to users about what data apps collect. Although this may make it more difficult for researchers to gather the data they need for their studies, it is also possible that these limitations will drive innovation in the field and lead to the development of new, more privacy-sensitive mobile sensing methods. This increased transparency is unlikely to provide a long-term impediment to mobile sensing research, as researchers already strive to be maximally transparent to participants about the data collected in their studies. The future of digital phenotyping and mobile sensing research will almost certainly be influenced by a trade-off between the requirement for precise and extensive data collection and the need to preserve individuals’ privacy and prevent unnecessary battery drain.

One of m-Path Sense’s shortcomings in comparison with other mobile sensing apps is the lack of a web-based dashboard in which the researcher can inspect and possibly extract all data at a glance. It should be noted that this functionality is already available for ESM data [26,45]. To accomplish this, a new pipeline (possibly with another backend) should be built, through which data are automatically extracted, imported, and stored in a structured format (as the R package does now). The researcher should then be able to generate several interactive plots to check the data via the web-based dashboard, such as a coverage plot (Figure 3).

When conducting mobile sensing research, it is not always necessary to collect and store all types of smartphone data. An important reason for this is to protect the participant’s privacy, but it is also because adding sensors consumes extra battery power, which is unnecessary. Therefore, one of our next steps forward will be to configure sensors and their sampling frequencies remotely. A concrete implementation could include allowing researchers to choose sensors from a web-based dashboard (as described previously) and adjust the sampling frequency according to their liking. This could also be done during the study if certain sensors are no longer considered to be relevant or if a sensor’s sampling frequency is found to be very high or very low.

A third important functionality for the future is the integration of event-contingent sampling, particularly regarding just-in-time adaptive intervention. CAMS already supports event-contingent sampling to some extent by allowing certain sensors to trigger each other. For instance, location activity could only be activated when the accelerometer activity increases, which saves battery when the participant is not moving. The web-based dashboard should be configured so that researchers can specify whether certain beeps or items should be requested only when a sensor reaches a certain value. A good example of this is in dyadic research, where beeps could only be requested when the participant is within Bluetooth range of their partner.

### Limitations

The study has certain limitations that should be considered when interpreting the results. A limitation is that the study was conducted as a pilot study with a small sample size; therefore, not all smartphone brands were adequately covered. This could have an impact on the validity of the findings and their generalizability to a large group. Another limitation is that mobile sensing technology is subject to changes and updates from companies such as Apple and Google; therefore, the results of this study are merely a snapshot of the performance at the time of measurement. At the same time, m-Path Sense is a software package that is continually evolving to stay up to date with these developments. These limitations should be considered when interpreting the findings; however, the study still provides useful information for evaluating the current performance of m-Path Sense.

### Conclusions

We combined the strengths of m-Path (a comprehensive ESM platform) and CAMS (mobile sensing) to produce m-Path Sense, a new mobile software app that prioritizes ESM and can be easily expanded to include mobile sensing. By examining its sampling reliability and perceived user experience in a pilot study, we found that the total amount of data gathered is sufficient for most studies, even though it is lower than the intended sampling frequency owing to OS limitations. Minor battery drain was reported by some individuals, but it was not considered to be problematic. m-Path Sense can be a step-up for research into digital phenotyping that calls for a combination of complete ESM and mobile sensing functionality, even though the accessibility of sensors is increasingly being restricted by OSs. Future studies should include a web-based dashboard for inspecting data and switching sensors on and off, event-contingent sampling, and more methods for monitoring and reactivating the app to minimize data gaps.

## Appendix

Multimedia Appendix 1 The average daily relative number of measurements across brands, where a value of 1 indicates that the actual number of measurements was exactly equal to the expected number of measurements and a value of 0.50 indicates that only half of the expected number of measurements were captured. ‘Other’ brands include included Nokia (n=2), Motorola (n=1), OPPO (n=1), Realme (n=1), and Xiaomi (n=1).

Multimedia Appendix 2 The average daily relative number of measurements across participants, where a value of 1 indicates that the actual number of measurements was exactly equal to the expected number of measurements and a value of 0.50 indicates that only half of the expected number of measurements were captured. While iOS often has a higher coverage rate, there are no obvious outliers, meaning that no participants (or groups of participants) consistently underperformed and lowered the average coverage rate. iOS: iPhone Operating System.

## Abbreviations

AES: Advanced Encryption Standard
CAMS: Copenhagen Research Platform Mobile Sensing
CRAN: comprehensive R archive network
EMA: ecological momentary assessment
ESM: experience sampling methodology
iOS: iPhone Operating System
OS: operating system
PKCS: Public-Key Cryptography Standard

## Data Availability

The data sets generated and analyzed during this study are not publicly available owing to privacy regulations related to the highly sensitive nature of the collected data but are available from the corresponding author upon reasonable request.
