# Supplementary figures and images for: Combining Experience Sampling and Mobile Sensing for Digital Phenotyping With m-Path Sense: Performance Study
Source: JMIR Form Res. 2023 Mar 7;7:e43296. doi: 10.2196/43296 (PMC10031448; doi:10.2196/43296)

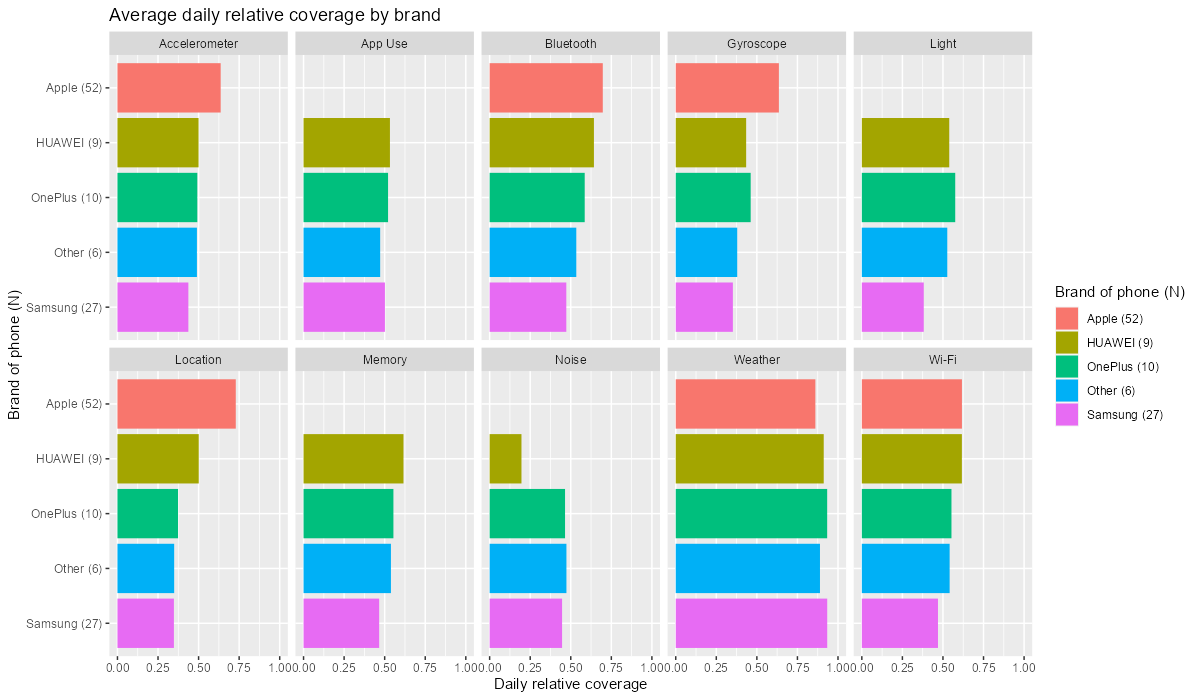

Supplement: Multimedia Appendix 1 [file formative_v7i1e43296_app1.png]

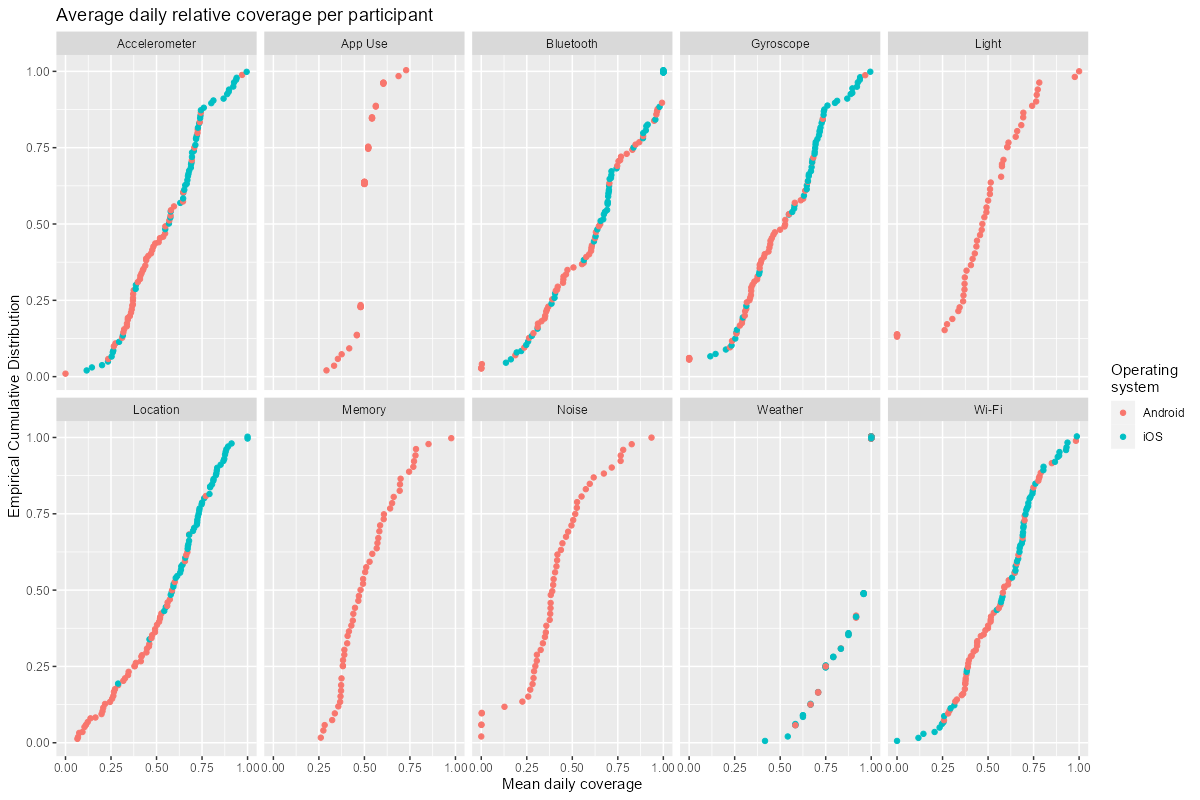

Supplement: Multimedia Appendix 2 [file formative_v7i1e43296_app2.png]
